# Antimicrobial use in agriculture: critical review of the factors influencing behaviour

**DOI:** 10.1093/jacamr/dlab178

**Published:** 2021-11-30

**Authors:** C McKernan, T Benson, S Farrell, M Dean

**Affiliations:** Institute for Global Food Security, School of Biological Sciences, Queen’s University Belfast, 19 Chlorine Gardens, Belfast BT9 5DL,UK

## Abstract

Antimicrobial resistance (AMR) is a global health emergency affecting humans and animals, diminishing the effectiveness of medication used to treat illness. The agri-food sector has attracted increased attention for imprudent antimicrobial use (AMU) and its contribution to AMR. Thus, ascertaining farmers’ and veterinarians’ behaviours surrounding AMU is essential to address imprudent AMU and generate behaviour change within the agri-food sector. Therefore, the aim of this critical review is to investigate, assess and collate the current body of evidence to identify psychosocial factors including knowledge, understanding, perceptions, attitudes and behaviours surrounding AMU. Database searches were limited to articles utilizing qualitative and quantitative methodologies, available in English with no restriction on publication year. Of the 1156 articles identified, 103 were retained for this review. Findings on the psychosocial aspects were thematically analysed. Five key themes emerged from the data: (i) knowledge and awareness of antimicrobials; (ii) attitudes towards antimicrobials; (iii) influential relationships; (iv) resources; and (v) factors influencing AMU. Results indicated that to overcome barriers experienced by key stakeholders, a carefully considered, evidence-based approach, incorporating behaviour change theory, is required when designing intricate interventions/strategies, in order to elicit successful and sustained AMU behaviour change.

## Introduction

Antimicrobials are essential to treat microbial infections and preserve the health of both humans and animals. However, imprudent antimicrobial use (AMU) accelerates the development of antimicrobial resistance (AMR), diminishing treatment efficiency and endangering the future of human and animal medical interventions.^1^^,^^2^ Typically, imprudent AMU refers to the overuse and misuse of antimicrobials in agricultural and healthcare settings, leading to environmental contamination, exacerbating AMR.^2^ International reports recognize AMR as a global health emergency, which if not addressed will cause serious health implications (such as prolonged illness and fatalities) and negative economic impacts.^2–4^ In response to AMR concerns, international frameworks have been developed to address the crisis such as WHO’s ‘Global Action Plan’ and the European Parliament’s ‘One Health Action Plan’.^2^^,^^4^ In summary, these reports recognize that a holistic multidisciplinary approach, across countries and organizations, is essential to combat imprudent AMU globally.

Historically, AMR in humans was attributed to imprudent AMU in human medicine. However, recently AMU in agriculture has come under the spotlight,^1^^,^^5^ with research reporting the potential to transfer AMR bacteria from food-producing animals to humans.^1^^,^^6^^,^^7^ While this connection is not fully understood, the discussions and uncertainty surrounding AMU in agriculture and its human health implications have focused research to address AMU within the sector.^1^^,^^8^

Antimicrobials have been used in agriculture for decades, with recent estimates revealing that 73% of all antibiotics worldwide are used in agriculture.^9^ Agricultural reliance on antimicrobials suggests habitual behavioural patterns, which are socially and culturally ingrained. Moreover, several reports indicate that antimicrobials are used imprudently in agriculture. For example, antimicrobials are frequently misused for growth promotion and prophylactic purposes. Additionally, high-priority critically important antimicrobials (HP-CIAs) that are primarily reserved for human treatment are used for animals.^8^^,^^10^^,^^11^ Due to the reliance on antimicrobials in agriculture, in 2019 the EU drafted regulations (EU 2019/6), prohibiting prophylactic AMU, which will come into effect from January 2022.

Interestingly, several reports have quantified country-specific sales of antimicrobials, highlighting that AMU varies significantly between countries.^9^^,^^10^ Moreover, recent reports on AMU within sectors have identified dairy farmers (mastitis) and pork farmers (weaning) as high antibiotic users.^12^^,^^13^ While these figures are beneficial and provide some insight on potential priority areas of concern, they do not provide a picture of what factors motivate the use of antimicrobials. Overall, changing behaviour has been identified as a crucial component by WHO and the EU to mitigate imprudent AMU.^4^^,^^8^ Consequently, in recent years there has been increased interest in understanding AMU in agriculture and more specifically, understanding farmers’ and vets’ knowledge, attitudes, perceptions and behaviours in relation to antimicrobials and AMR, to understand factors influencing decision making and current practice.^14^^,^^15^

Interestingly, the published literature, while varied in aims and research design (qualitative and quantitative), has identified that factors such as knowledge, attitude and access to resources can influence agricultural AMU behaviour and help to motivate behaviour change, mitigating AMR.^14–17^ Therefore, the aim of this critical review is to investigate, collate and integrate the current body of evidence pertaining to key stakeholders (farmers and vets), in relation to knowledge, understanding, perceptions, attitudes and behaviours surrounding AMU and AMR. This will allow for the identification of barriers and facilitators in relation to AMU and identify research gaps. Understanding barriers and facilitators will inform strategies to promote and sustain behaviour changes, mitigating imprudent AMU in agriculture, and will advise the direction of future behavioural research in relation to AMU.

## Materials and methods

### Search strategy

This critical review was completed in a structured approach in accordance with the preferred reporting items for systematic reviews and meta-analysis (PRISMA) guidelines, via the completion of several key steps.^18^ In order to satisfy the research question, articles exploring farmers’ and vets’ knowledge, attitudes, perceptions of antimicrobials and AMR were sourced. Initially, search terms were generated to cover the scope of the research aims. To ensure that the author had exhausted the relevant terminology, the second author (T.B.) reviewed the search terms identifying three additional terms. Search terms involving stakeholders of interest included *farmers, producers, vet, veterinarian* and *agriculture*. Additional searches were conducted involving different sectors of interest such as *livestock, pigs, swine, porcine, bovine, ruminant, dairy, cow, beef, sheep, chicken, poultry* and *flock*. To address the psychological aspect, terms such as *behaviour, opinions, attitudes, beliefs, perceptions, knowledge, barriers, facilitators, understanding, thoughts* and *feelings* were included. Literature searches for antimicrobials involved terms such as *antimicrobial, antimicrobial resistant, antibiotic, antibacterial* and *antibacterial resistant.* In September 2020, a comprehensive and systematic search of the keywords catalogue was conducted across four electronic databases: MEDLINE, PsycINFO, Scopus and Web of Science.

### Screening

**Figure 1.**
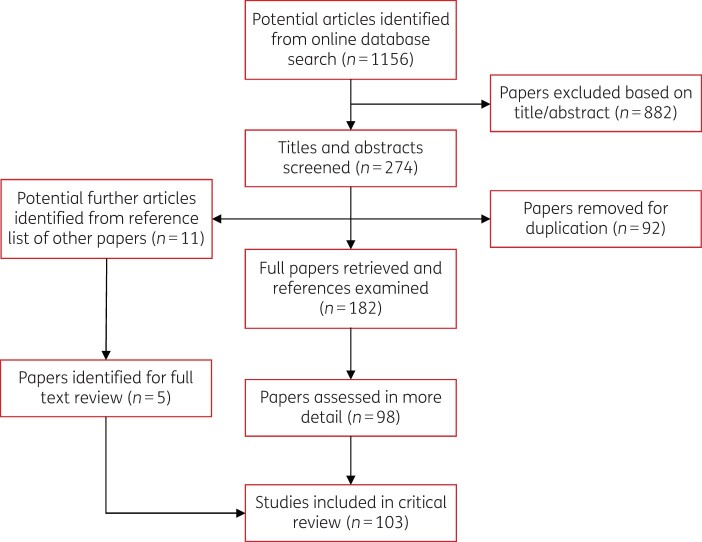
Flow diagram illustrating the assessment and selection of articles for review.

Initially, 1156 papers were retrieved from the database searches. One author (C.M.K.) independently screened article titles and abstracts. Duplicated articles were crosschecked and removed. Overall, 274 relevant articles were sourced based on the title and abstract. Subsequently, utilizing the eligibility criteria (provided below) a total of 98 papers were selected. A further five articles were identified from manually searching reference lists for key articles of interest. As a result of this approach, a total of 103 papers were sourced for full-text review (see Figure 1).

### Eligibility criteria

The eligibility criteria were as follows:

- Stakeholder: veterinarian, farmer, or both.
- Sectors: ruminant (dairy/beef), porcine, poultry, sheep.
- Study design: qualitative and quantitative approaches including, interviews, focus groups, questionnaires or mixed methods.
- Outcomes: report on themes, areas of interest in relation to the knowledge, perceptions, attitudes and behaviour surrounding antimicrobials.
- Language: published in English.
- Date: no restriction on the year of the study or publication.
- Location: no restriction on region.

### Data extraction and synthesis

All articles were analysed, and the following data were extracted: country of study, stakeholder, sector, study design, sample size, strengths, limitations, key results and outcomes. Extracted data were thematically coded inductively in accordance with the Braun and Clarke protocol.^19^ Findings from the eligible articles were coded for information of relevance to satisfy research aims in relation to farmers’ and vets’ knowledge, attitudes and perceptions of antimicrobials and AMR. From the generated codes, five key themes were constructed and are described in the results. To ensure the reliability of the sample, 50% of papers that were independently reviewed were crosschecked and verified by a second author (T.B.). A further 10% were reviewed by the third author (M.D.) to ensure consistency in the data extraction approach.

## Results

### General results

**Figure 2.**
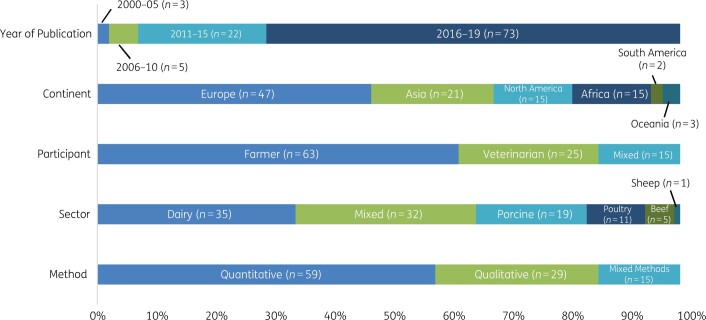
Characteristics of articles included in critical review.

The 103 articles relevant for this critical review were published between 2002 and 2020, with the majority (>50%) published between 2016 and 2020. While research was conducted in 48 countries globally, the majority of the research (46%) was concentrated in European countries. Furthermore, survey questionnaires (57%) were the most popular study design, followed by interviews (28%) and mixed methods (15%). Sectors most frequently researched (in descending order) were dairy ruminants, porcine, poultry, beef and sheep (Figure 2).

Five key themes relating to (i) knowledge and awareness of antimicrobials, (ii) attitudes towards antimicrobials, (iii) influential relationships, (iv) resources and (v) factors that influence AMU were identified. Please see Table S1 (available as Supplementary data at *JAC-AMR* Online) for a comprehensive summary of key themes and sub-themes identified in this review. Here the authors have recorded the number of articles investigating each theme, differentiating between methodologies and providing interpretive quotes.

### Theme 1: Knowledge and awareness of antimicrobials

#### Farmers’ knowledge and awareness

Farmers in Africa (Cameroon, Ghana, Ethiopia, Nigeria, Sudan, Tanzania and Uganda), South America (Peru) and Asia (Bangladesh, Cambodia, China, India, Indonesia, Malaysia, Thailand and Vietnam) exhibited limited knowledge of the purpose of antimicrobials and the consequences of imprudent AMU i.e. AMR.^20–25^ Additionally, farmers in Sudan and Peru were unaware of regulations surrounding AMU i.e. withdrawal periods.^23^^,^^26^^,^^27^ In comparison, farmers in Europe (Belgium, Denmark, France, Germany, Italy, the Netherlands, Spain, Sweden, Switzerland, Turkey and the UK), the USA (New York, Michigan, Tennessee and South Carolina) and Canada demonstrated an enhanced knowledge of antimicrobials and AMR.^17^^,^^28–34^ Farmers in these countries were knowledgeable about the purposes of antimicrobials, were aware that good farm management practices mitigated disease occurrence and transmission, and appeared coherent in choice of treatment.^15^^,^^18^^,^^31^^,^^32^^,^^35–39^ Nevertheless, while farmers in these countries displayed a decent understanding of AMU and AMR, some confusion and misconceptions remained, particularly in relation to the consequences of imprudent AMU i.e. AMR.^16^^,^^30^^,^^33^^,^^38–42^ For example, a UK study reported that 86% of farmers considered themselves knowledgeable regarding AMR; however, only 55% provided an accurate description of it.^40^ Another UK study identified that despite perceived high knowledge and concern surrounding HP-CIAs, many farmers could not identify them.^16^^,^^43^

Studies demonstrated that knowledge also varied with age, with younger farmers in general displaying a greater knowledge of antimicrobials and being more likely to consider the impact of AMR in treatment choice.^32^^,^^33^^,^^44–48^ However, in Nigeria, younger farmers were less knowledgeable about antimicrobials in comparison to older farmers.^49^ Similarly, in five African countries (Tanzania, Kenya, Zimbabwe, Zambia and Ghana), increased years’ experience correlated with increased knowledge and attitudes towards prudent AMU.^46^ Interestingly, Nigerian females were more knowledgeable about antimicrobials than Nigerian males, whilst males were more knowledgeable than females in China.^44^^,^^50^ Additionally, farmers with higher education levels were also more likely to possess satisfactory knowledge about antimicrobials.^32^^,^^44^^,^^49^ Meanwhile, in Wisconsin (USA), Malaysia, India and Peru, larger farm enterprises were more knowledgeable about preventative and responsible practices, perhaps making them better equipped to implement strategies in comparison to smaller enterprises.^23^^,^^51–53^ Contrastingly, in the UK and Sweden, larger herd sizes were associated with increased reliance on antimicrobials.^40^^,^^45^

Globally, elevating farmer knowledge through education was found to influence AMU and encourage appropriate practices on farms across all sectors, countries and regions.^20^^,^^26^^,^^32^^,^^39^^,^^44^^,^^45^^,^^48^^,^^54–58^ Moreover, it is important to note that farmers were receptive to interventions that improved knowledge and awareness. For example, in the UK, Italy and Germany, farmers were interested in strengthening their knowledge. They expressed interest in attending training courses in conjunction with fostering veterinary collaboration, attempting to create an environment to share success stories encouraging behaviour change. Overall, providing farmers with the necessary confidence, skills and support has been shown to enable the transition to reduce reliance on antimicrobials, without negatively affecting production.^17^^,^^29^^,^^40^^,^^41^^,^^59^ Similarly, in Cameroon, Tennessee, Indonesia and Vietnam, additional training was supported by farmers.^21^^,^^39^^,^^57^^,^^58^^,^^60^ In Peru, increased knowledge of antimicrobials was associated with using them in a preventative manner.^23^ While, in the UK and Tennessee, affiliation with assurance schemes or herd health plans, capable of providing training opportunities, improved compliance with antimicrobial stewardship recommendations, in addition to increased market access.^16^^,^^31^^,^^37^ In France, Norway and Nigeria, farmers and vets use discussion-based platforms or collaborative workshops to promote knowledge exchange and provide practical skills to reduce AMU.^36^^,^^41^^,^^55^ Meanwhile, recently (2017) in Thailand a national global action plan (GAP) was implemented to combat AMR. The GAP was found to elevate farmers’ knowledge, concern and attitude towards strategy implementation, in comparison to the non-GAP group, demonstrating an effective approach that could be used to motivate farmers to adopt and implement antimicrobial stewardship strategies.^61^

#### Vets’ knowledge and awareness

Several studies found that European vets were thoroughly aware that imprudent AMU (i.e. overprescribing) contributed to AMR; in addition to being knowledgeable of the recommended practices and policies to reduce the need for antimicrobials.^17^^,^^61–67^ However, a Dutch study indicated that veterinary knowledge surrounding antimicrobials required improvement.^68^ In India, vets were only partially aware of the risks of imprudent AMU, illustrating gaps in their scope of knowledge.^69^^,^^70^ However, in Africa and Vietnam, the majority of vets had a high awareness of AMR.^71^

Interestingly, Dutch vets believed educational programmes should be made compulsory for farmers,^68^ while other research suggested that providing education for vets focused on AMU would promote a harmonized approach for prescribing.^72^^,^^73^ In the UK, vets suggested that farmer workshops or discussion groups were an important strategy to encourage stewardship, facilitating vet and farmer collaboration to share positive experiences and discuss strategies; thus motivating other farmers to make changes.^17^^,^^74^ Swiss vets reported that participation in peer study groups enables continuous education and can change the mindset and intention of vets in a positive way.^75^ Generally, vets believed continuous education, through training courses, was the most beneficial and effective approach to simultaneously increase knowledge and improve AMU behaviour.^59–62^

Overall, the level of knowledge and awareness of antimicrobials and AMR amongst farmers fluctuates hugely. It is evident that farmer knowledge and awareness in developing countries was low, while there was confusion and misunderstanding in developed countries. This highlights the substantial differences in educational campaigns and support provided by different governments. For example, in countries such as Denmark, Switzerland, Norway and the UK, individual strategies have been launched focusing on providing education and training programmes, thus elevating knowledge and practices.^16^^,^^76^^,^^77^ Comparatively, in other countries such as Thailand, only recently (2017) was a national GAP launched to support farmers to combat AMR.^61^ Unsurprisingly, vets were knowledgeable on the purpose of antimicrobials and the potential risks. Overall, studies have ascertained that elevating knowledge is one way to instigate behaviour change,^78–80^ with numerous studies highlighting the benefits of different platforms used for farmers and vets to increase awareness of antimicrobials and compliance with antimicrobial stewardship strategies. Therefore, future strategy design should facilitate continuous education opportunities suitable for vets and farmers, which will elevate their knowledge.

### Theme 2: Attitudes towards antimicrobials

#### Farmer and vet responsibility

In Norway, Germany, Sweden and Tennessee, preserving the health and welfare of animals is considered an important characteristic of being perceived as a ‘good farmer’. Furthermore, farmers described an emotive bond to livestock and therefore felt a responsibility to maintain herd health.^15^^,^^17^^,^^38^^,^^54^^,^^55^^,^^81–83^ Similarly, numerous UK studies indicated that vets felt a sense of responsibility in ensuring animal welfare and alleviating animal suffering.^17^^,^^43^^,^^64^^,^^74^ Norwegian farmers indicated that maintaining herd health without depending on antimicrobials gave farmers a sense of personal satisfaction and control.^55^ In the Netherlands and Germany, farmers extended treatments to alleviate the guilt of disease recurrence and saw this as a socially accepted behaviour of a ‘good farmer’.^82^ Furthermore, in the Netherlands and the UK, vets felt obligated to treat sick animals in circumstances where the disease could have been prevented if the farmer had implemented the vet’s advice.^64^^,^^68^ Vets also reported that they have a dual responsibility: provide advice and preserve animal welfare and maintain the economic stability of the farmer.^68^^,^^74^ Additionally, vets admitted that ultimately the farmer makes the final decision as to whether preventative measures and administration instructions are adhered to or not.^68^ Conversely, farmers in South-East Asia did not believe they were responsible for ensuring prudent AMU,^20^^,^^28^^,^^84^ while the majority of farmers in New Zealand and the UK placed the main responsibility for monitoring prudent AMU with the vet.^17^^,^^85^^,^^86^ Similarly, in the UK, both vets and farmers placed the onus of ensuring responsible antimicrobial practices with the vet.^17^^,^^35^

#### Farmer and vet optimism bias and misconceptions

Optimism bias is a cognitive bias causing an individual to underestimate the possibility of a negative event in the future whereby they believe that they themselves are less likely to experience a negative event.^87^ Research discovered that Indonesian farmers displayed optimism bias, as they found that farmers believed that that AMR would not be an issue on their farms.^21^

Conversely, numerous studies illustrated that farmers and vets hold certain misconceptions regarding their contributions to AMR. For example, although the vast majority of vets acknowledged that irresponsible AMU contributes to AMR, American, Australian, Dutch and UK farmers doubted the extent to which AMU in agriculture resulted in AMR and risks to human health.^17^^,^^30^^,^^35^^,^^68^^,^^88^^,^^89^ Similarly, vets in Australia considered AMU in agriculture as a moderate concern and contributor to AMR,^88^ despite 65% of vets observing treatment failure.^89^ Finally, UK and American farmers plus UK and Australian vets believed that other agricultural sectors and specifically human medicine were more accountable for AMR contribution; furthermore, considering themselves as prudent users of antimicrobials.^17^^,^^31^^,^^34^^,^^35^^,^^39^^,^^51^^,^^64^^,^^83^^,^^88^

In a similar vein, studies in Belgium, France, Germany, the Netherlands, Sweden, Switzerland and the UK reported that farmers considered their own AMU to be lower in comparison to farmers in other countries and sectors, illustrating that they had removed themselves as contributors to AMR.^17^^,^^29^^,^^59^^,^^82^^,^^85^^,^^89^^,^^90^ Similarly, in India, vets believed that over-prescription of antimicrobials exacerbated AMR and claimed they used antibiotics judiciously, attributing AMR occurrence to farmers’ ‘quacks’ and paravets.^65^^,^^69^ Whereas, in Canada and New Zealand, although farmers were concerned about AMR, less than half of the farmers considered AMR when deciding on treatments, demonstrating a lack of personal accountability.^33^^,^^86^ Likewise, in Italy, some vets believed that new antimicrobials had been developed and are available to replace those of diminished effectiveness.^62^ Worryingly, in Uganda, while 65% of farmers believed that they used antimicrobials in accordance with recommended guidelines, limiting their contributions to AMR, only 16% complied with recommendations.^56^

#### Farmers’ and vets’ attitudes toward disease risk and antimicrobial stewardship strategies

##### Disease risk

European studies found that farmers’ attitudes towards regulations, risk perception and perceived requirement of antimicrobials varied considerably between sectors^48^ and countries.^91^ In Ohio and New York, production type influenced risk perception, with organic farmers showing higher AMR concern and greater consideration to recommended alternative practices compared with conventional farmers.^34^^,^^47^ Numerous European studies reported that farmers’ individual attitudes, habits and perceived risks and benefits towards AMU and AMR influenced practices, in comparison to grouped livestock sectors.^38^^,^^48^^,^^89^^,^^91^ Similarly, in Cameroon, strong correlations between risk perception of AMR with individual practices, attitudes and knowledge were evident.^57^ Many farmers referred to disease risk on their farm with colloquialisms such as ‘touching wood’, ‘keeping fingers crossed’ and ‘bad luck’.^37^^,^^92^

##### Antimicrobial stewardship strategies

Studies in North America and Europe strengthen the finding that farmers’ individual perceptions and attitudes towards reduction strategies are fundamentally important and are based on personal evaluation factors including uncertainty of animal recovery, financial consequences, knowledge, perceived feasibility, effectiveness of the strategy and perceptions of negative consequences.^37^^,^^38^^,^^83^^,^^89^^,^^93^^,^^94^ Likewise, several studies report that vets’ initial perceptions of, and attitudes towards, the complexity, feasibility and effectiveness of strategies focused on reducing AMU influenced success and implementation.^63^^,^^73^^,^^74^^,^^76^^,^^88^^,^^95–98^ European studies reported that farmers and vets with lower perceived need for antimicrobials, coupled with stronger beliefs about the feasibility and effectiveness of a strategy, correlate with an increased intention to reduce AMU on the farm and a more positive experience with implementation.^89^^,^^94^^,^^97–99^ However, in the UK, only 26% of farmers would promote strategies to other farmers, stressing that if it went wrong, they would feel that they would be blamed.^98^ Scherpenzeel *et al.* (2018)^73^ reported that groups of vets who had neutral or unfavourable attitudes towards stewardship strategies felt pushed to comply, subsequently leading to a negative experience.

Hektoen (2004)^55^ reported that Norwegian farmers favoured homeopathic treatments in comparison to antimicrobials, emphasizing that scientific evidence was not essential, as they had experienced benefits. In relation to attitudes to stewardship strategies, in Thailand, favourable attitudes towards strategy implementation correlated with the increased knowledge of the national GAP scheme.^61^ In contrast, in the USA, when the Veterinary Feed Directive (VFD) was launched to regulate and reduce AMU, attitudes towards this programme were variable with many farmers disliking the VFD, considering it an unnecessary and burdensome requirement that brought additional costs and interfered with their business.^34^^,^^60^ In New Zealand, the vast majority of vets felt that policies outlined by the Veterinary Association were feasible and implementation would reduce AMU.^86^

Meanwhile, several European studies reported that awareness and knowledge of antimicrobial stewardship recommendations do not imply farmers will implement or comply.^16^^,^^36^^,^^37^^,^^40^ For instance, in the UK, it was found that 53% of respondents were aware of Responsible Use of Medicines in Agriculture Alliance (RUMA), and only 36% of these followed guidelines properly.^16^ Similarly, in France and the UK, despite farmers’ enthusiasm in relation to the Farm Register and RUMA, these strategies were associated with variable levels of farmer compliance, complaints about copious paperwork and inspections and lack of financial return.^36^^,^^37^ These studies suggest that while knowledge and awareness of interventions and strategies is an important element of behaviour change, it is not enough on its own to motivate behaviour change. European studies indicated that interventions should be country- and sector-specific and address individual barriers, optimizing the benefits of reducing AMU.^29^^,^^82^^,^^100^ Similarly, several farmers and vets stressed that interventions should be tailored so that they are sector-specific, as perceived efficiency and feasibility of interventions are crucial for the intervention to succeed.^66^^,^^76^^,^^91^^,^^96^^,^^101^ Farmers in the USA reported that reducing AMU would be more efficient if standardized methods to record treatments were developed and implemented.^51^

#### Farmers’ and vets’ attitudes towards reducing AMU

Vets in Australia, Germany and Ohio were concerned about the effects of AMR on animal and human health.^59^^,^^63^^,^^88^ Similarly, European farmers were concerned about AMR and believed that AMU can be reduced.^42^^,^^59^^,^^90^ However, in China and other parts of the USA, farmers did not appear to be as concerned.^34^^,^^39^^,^^50^ Numerous European countries, including Norway, Italy, the Netherlands, Germany and the UK, recognized their own responsibility to combat imprudent AMU with the majority (>70%) of farmers emphasizing their desire to or actively trying to implement strategies to reduce AMU.^16^^,^^17^^,^^29^^,^^40^^,^^43^^,^^55^^,^^82^^,^^94^^,^^99^ Similarly, the majority of Dutch and Australian vets supported the reduction of AMU in livestock, believing they could be a ‘good vet’ by reducing the prescription of antimicrobials.^73^^,^^76^^,^^88^ Farmers reported that reducing reliance on antimicrobials is beneficial for various reasons, including lowering costs (treatment and veterinary consultation) and increasing profit, while simultaneously improving consumer confidence in produce and marketability.^16^^,^^40^^,^^44^^,^^55^^,^^82^^,^^83^^,^^94^ Interestingly, Spanish pig farmers indicated that they would be unable to reduce AMU on farms.^102^

Farmers in Asia, Africa, the USA and Spain believed it was beneficial and justifiable to routinely use antimicrobials for financial benefits, including improving production, maintaining herd health, disease prevention and reduced mortality rates.^22^^,^^23^^,^^28^^,^^39^^,^^44^^,^^51^^,^^54^^,^^58^^,^^60^^,^^102–104^ This belief was echoed by vets in Cambodia believing that livestock would not thrive without the routine application of antimicrobials.^104^ Farmers in Europe and the USA worried that reduced AMU would compromise animal health and welfare,^16^^,^^17^^,^^34^^,^^35^^,^^67^^,^^98^^,^^105^ with farmers and vets in the UK and Switzerland expressing concern that further legislative restrictions prohibiting AMU would threaten their future ability to treat sick animals effectively, and thus compromise animal welfare and threaten livelihoods.^17^^,^^67^ In the UK, the majority (>80%) of farmers were interested in or actively trying to implement strategies to reduce AMU. However, it was recognized that farmers did not feel equipped with the technical knowledge and skills to effectively implement strategies without detrimentally impacting production.^16^^,^^40^^,^^41^^,^^85^ In the UK and Canada, despite farmers’ interest in reducing AMU, they were hesitant to implement strategies due to economic concerns relating to the loss of stock, contracts and profit.^33^^,^^98^ Moreover, European farmers and vets favoured AMU and were wary of change, believing the benefits of using antimicrobials outweighed the risks.^17^^,^^97^^,^^98^ Interestingly, whilst farmers and vets in the UK displayed an increased awareness and willingness to combat AMR, these stakeholders also considered AMR as a future threat, meaning that AMR was frequently dismissed in the face of more immediate concerns and challenges on the farm.^17^^,^^35^ Vets expressed concern that the reduction of antimicrobials may not be economically viable or practically feasible, and coupled with farmer reluctance could adversely affect animal welfare, increase fatality and damage the vet’s reputation.^17^^,^^64^^,^^74^ Furthermore, some vets who had a negative experience with antimicrobial stewardship strategies were reluctant to encourage such strategies to other farmers and vets.^74^

Overall, the challenge of changing attitudes in European countries is complex; where knowledge and awareness are higher, farmers and vets perceive themselves as prudent users of antimicrobials and blame other sectors and stakeholders for contribution to AMR, rather than themselves. Therefore, they are not motivated to change behaviour, as they do not consider their behaviours to contribute to AMR. Conversely, farmers and vets in developing countries rely on AMU for animals to thrive, and it is a social norm to use antimicrobials freely. In developed countries, despite the desire to reduce AMU, vets and farmers were concerned about the repercussions of reducing AMU, owing to feelings of fear and uncertainty that reducing AMU would diminish animal welfare and productivity. Moreover, farmers’ and vets’ individual perceptions towards disease risk and stewardship approaches are vital when it comes to the successful implementation of an antimicrobial stewardship strategy. Therefore, strategy design should be tailored to meet the needs of specific sectors and regions. Furthermore, these strategies should focus on improving numerous psychological aspects such as knowledge, confidence and self-efficacy, which will improve overall attitudes towards AMU and help to successfully promote AMR reduction.

### Theme 3: Influential relationships

#### Vet–farmer relationship

It is evident from worldwide research that vets are considered the primary advisor providing farmers with the most valuable, credible, trustworthy and relatable advice.^15–17^^,^^29^^,^^33^^,^^35–37^^,^^41^^,^^59^^,^^82^^,^^86^^,^^91^^,^^102^ Vets acknowledged that farmers follow and trust veterinary advice. However, they indicated that this was dependent on how the farmer perceived the effectiveness and expense of the approach.^63^^,^^64^^,^^72^^,^^95^^,^^97^ Dutch studies recognized that vet encouragement and support are fundamentally important, as they can influence farmers’ perceptions, resulting in the successful implementation of antimicrobial stewardship strategies i.e. selective dry cow therapy.^68^^,^^73^ Furthermore, increased vet farm visits facilitated communication and promoted collaboration; this in turn enhanced farmer knowledge and awareness of responsible AMU and AMR and improved compliance with recommended AMU reduction strategies.^17^^,^^36^^,^^38^^,^^40^^,^^41^^,^^44^^,^^97^ Interestingly, Norwegian farmers did not consider vets as primary discussion partners in relation to homeopathic treatments, instead only seeking veterinarian consultation if homeopathic approaches were unsuccessful or the disease was considered severe.^55^ Vets in France, the UK and the Netherlands indicated that in addition to their therapeutic role, vets are increasingly focusing on providing farmers with pragmatic advice promoting antimicrobial stewardship; thus promoting disease prevention without adversely affecting production.^17^^,^^68^^,^^72^^,^^101^^,^^106^^,^^107^ Conversely, in India, vets were unaware that the provision of advice to use good farm management practices was an integral part of their role, rarely discussing previous experiences with clients.^65^ Overall, numerous studies indicated that strategies focused on this important stakeholder relationship between the vet and farmer are fundamentally important to facilitate change and improve herd health, as farmers sought veterinary guidance and support to motivate change.^17^^,^^76^^,^^96^^,^^97^

Vets in the Netherlands and the UK acknowledged that it is difficult to engage and motivate behaviour change in farming practices due to risk avoidance, uncertainty, insufficient knowledge and perceived benefits for AMU.^63^^,^^68^^,^^74^^,^^76^ Vets believed that farmers are less concerned with the diagnosis and more concerned with receiving treatment.^72^ Moreover, a cross-European study found that vets were more optimistic and enthusiastic about reducing AMU in comparison to farmers.^97^ Farmers’ personalities also influenced the willingness of vets to raise the topic of antimicrobial stewardship and discuss alternative drugs or preventive measures; similarly, there was frustration at those farmers who appeared reluctant to change.^17^^,^^74^ However, in the UK, vets recognized that they may have a pre-existing flawed perception that is it difficult to change farmer behaviour, realizing that is it important to engage farmers and understand their needs, to enable communication and promote positive change.^17^ In Peru, vets acknowledged their clients had a low knowledge level, making it difficult to help those farmers understand prescribing practices.^108^

#### Peer support and previous experience

##### Farmers

In countries such as Sudan, Ghana, India, Peru, Thailand, Central America and Vietnam, farmers are dependent on sharing experiences with peers to acquire knowledge and practices.^23^^,^^24^^,^^26^^,^^50^^,^^102^^,^^109–112^ In Uganda, indigenous homeopathic therapies were relied upon,^56^ while farmers in Africa and Asia decided on treatment based on their own experiences.^44^^,^^113–115^ In European countries, farmers’ previous experience and sharing experiences with peers remains a trusted and relatable knowledge network frequently favoured over scientific evidence-based advice.^16^^,^^30^^,^^32^^,^^37^^,^^39^^,^^54^^,^^55^^,^^81^^,^^82^

##### Vets

Vets in Australia, New Zealand, Germany, the UK and Denmark depend on previous experience or the advice of peers when prescribing antimicrobials.^15^^,^^59^^,^^64^^,^^86^^,^^88^^,^^116^^,^^117^ In Ohio, New Zealand and Nigeria, knowledge surrounding AMR was negatively correlated with increasing veterinary experience.^63^^,^^86^^,^^118^ Furthermore, studies completed in the Netherlands and Ohio reported more experienced vets were less concerned about the consequences of AMR, with less experienced vets being more receptive, optimistic and proactive about implementing antimicrobial stewardship approaches to reduce AMU.^63^^,^^76^ Additionally, more experienced vets were less hesitant and more confident in the diagnosis and choosing antimicrobial treatments for livestock.^76^^,^^106^ Therefore, a less experienced vet’s lack of confidence, coupled with the difficulty in gaining a farmer’s trust, inhibits independence and involvement when selecting the treatment approach.^64^^,^^74^ Some vets admitted they occasionally prescribed against their own judgement, as they suspected their colleagues would override their decision, undermining their relationship with their farmer clients.^17^ Irish and Dutch vets suggested that each farmer should be assigned one vet with routine visits, to promote good farm management practices and a consistent treatment approach, in addition to avoiding conflicts from other farmers and colleagues.^76^^,^^101^

##### Drug vendors

It is important to note that farmers in developing countries relied on information provided by local drug vendors.^44^^,^^58^^,^^108^^,^^110^ Moreover, farmers in developing countries have requested that drug store vendors should be certified, regulated and monitored.^44^^,^^58^^,^^109^^,^^111^^,^^113^

#### Government and society

Several studies have acknowledged a fractured relationship between society, government bodies and farmers,^15^^,^^37^^,^^82^ with numerous studies reporting that farmers felt frustration and a lack of appreciation from society through the media for their work and antimicrobial stewardship efforts.^15^^,^^17^^,^^82^ Farmers in the UK and South-East Asia felt unsupported by government bodies, frequently describing government strategies as impractical.^17^^,^^37^ In the UK and Switzerland, vets believed political pressure to change legislation to further restrict AMU originated from poor public awareness and perception of agriculture’s AMU rather than scientific evidence.^17^^,^^35^^,^^64^^,^^67^ Unsurprisingly, vets hold the belief that scientific evidence should be used to change policies rather than social pressure.^35^^,^^68^^,^^88^^,^^89^ In the UK, some farmers believe that AMU is intrinsically associated with certain farm systems and feel powerless to invest in farm structures, feeling that consumer demand for cheap produce promotes intensification and poorly managed systems. In the UK and the USA, farmers believed that misconceptions existed with consumers about AMU in agriculture and felt that consumers believed raising animals without antibiotics would significantly improve animal welfare. Subsequently, farmers believe consumer misconceptions and marketing strategies are driving regulations, thus endangering agricultural sustainability in the future.^34^^,^^43^^,^^105^ In the USA, this consumer misconception of AMU in agriculture has become a reality, leading to a market-driven scheme for animals to be raised without antibiotics (RWA). Vets and farmers involved in the scheme admitted that maintaining the RWA status was prioritized over animal welfare.^104^ In the UK, farmers recognized the potential of consumers to drive improvements in AMU and welcomed supermarket and industry-led initiatives centred around financial support (via better prices for produce) to drive continuous improvement on farms.^17^

In European countries, the relationship between vets and farmers is particularly important when considering strategy design to reduce AMU. Future interventions should foster this collaborative relationship, which allows both farmers and vets to take ownership of antimicrobial stewardship strategies, as it is perceived as a joint enterprise. This platform allows farmers and vets to share knowledge, success stories and experiences to support farmers’ transition to reduce AMU. Furthermore, studies in clinical settings have observed favourable outcomes when training clinical staff in motivational interviewing, with it promoting improved communication between client and patient; ultimately resulting in increased client and patient satisfaction and team cohesion.^119^^,^^120^ Training vets to utilize this approach may improve understanding and communication in this instrumental relationship. Comparatively, in developing countries, farmers have limited access to professional services and therefore depend on peers, previous experience and drug vendors for information and advice. Due to the reliance on vet drug-shop owners, interventions should target improving the knowledge of this stakeholder in these regions to harmonize treatment choices.^111–113^

Overall, the fractured relationship between the government, consumers and agriculture is evident. In addition, the dangers of consumer misconceptions driving marketing strategies and policies are becoming evident, as the RWA scheme in America has shown.^102^^,^^118^ Therefore, it is important that the message of AMU in agriculture is communicated carefully with consumers; namely that agricultural AMU needs to be reduced but not completely eliminated, as this will have negative ramifications for animal welfare.

### Theme 4: Resources (Economic and information)

#### Economic

While European farmers and vets recognized the importance of implementing AMU reduction strategies, such as upgrading the farm environment (by modernizing buildings, promoting vaccinations and improving stockmanship), farmers also reported that a combination of structural restrictions, time and economic constraints inhibits their ability to invest in this, to improve herd health and alleviate AMU.^15^^,^^17^^,^^81^ Some farmers indicated that AMU was a ‘management tool’ to compensate for the inability to invest.^43^ Similarly, vets recognize that farmers struggle to invest due to limited finances, coupled with the difficulty in predicting the cost-effectiveness of specific measures on individual farms. This makes it difficult to convince farmers to implement specific measures, as economic considerations are a major driver for decisions made on farms.^17^^,^^56^^,^^64^^,^^68^^,^^76^^,^^97^ Likewise, in Asia and Africa, the challenges of economic vulnerability, tight profit margins, scarcity of funding facilities and limited finance for veterinary consultations, coupled with substandard farming structures and practices and poor infrastructure, contribute to AMU reliance to preserve productivity.^22^^,^^24^^,^^27^^,^^28^^,^^44^^,^^46^^,^^49^^,^^69^^,^^104^ Furthermore, in the UK, farmers and vets acknowledged the short-term low cost of using antimicrobials was more amenable than high-cost investment in farm management practices.^35^^,^^43^^,^^99^ Interestingly, however, in a UK pig study, farmers were motivated to use alternative approaches due to the high cost associated with AMU e.g. vaccination protocols to alleviate disease occurrence, primarily to balance the economic cost of disease and improve profitability.^43^

Overall, European and American vets were sympathetic to the financial pressures and constraints faced by farmers, with both farmers and vets acknowledging that strategies surrounding financial implications, such as compensation, endorsement of vaccination programmes, bonuses to invest in the farm environment and financial penalties would be effective approaches to mitigate imprudent AMU, promote farmer involvement, facilitate disease prevention and reduce AMU.^24^^,^^37^^,^^44^^,^^68^^,^^81^^,^^83^^,^^90^^,^^101^^,^^121^ This emphasizes that strategies to reduce AMU should be incentivized by financial reward as opposed to financial punishment.^81^^,^^90^

In terms of veterinarian consultation, although it is a trusted source of information, farmers in the UK and the USA felt that it did not have a significant role in improving antimicrobial stewardship on-farm, due to high costs; therefore reduced veterinary tariffs would encourage use.^17^^,^^34^^,^^83^ Similarly, farmers in Nigeria believed that vets should make their services more affordable and accessible.^44^ Proceeding to the examination of veterinary profit from the sale of antimicrobials, remarkably farmers in the Netherlands, Germany, Switzerland and the UK did not associate vet chosen treatment with financial gain and felt that decoupling prescribing and dispensing would have little effect on AMU, further demonstrating the trust in this important relationship.^43^^,^^82^^,^^89^ Numerous European and Australian vets echoed farmers’ judgements, strongly emphasizing that the potential for increased revenue when prescribing antimicrobials did not influence treatment choice. Therefore, decoupling antimicrobial sales from vets was perceived as an ineffective approach to reduce AMU.^64^^,^^68^^,^^76^^,^^88^^,^^116^

Interestingly, in France, there is inconsistent financial support within sectors for diagnostic tests. The porcine and poultry sectors receive financial support while the bovine sector does not.^72^ Similarly, in India, the uptake of vaccination programmes was region specific and based on financial relief.^69^ In contrast, research completed in six European countries suggested that interventions involving financial incentives or penalties were not considered a motivator for antimicrobial stewardship activities.^97^

#### Information sources

In addition to the farmers’ relationships with vets, drug vendors and peers, other common sources of information with variable weights of influence and credibility included nutritionists, regulators, governmental policymakers, magazines, pharmaceutical companies and scientific evidence.^16^^,^^30^^,^^33^^,^^39^^,^^58^^,^^59^^,^^82^^,^^83^^,^^109^ Vets considered knowledge provided through peers and previous experience as a source of information. Research has also identified other sources of information including journal publications, government recommendations, policies and legislation.^63^^,^^100^^,^^116^ In Tennessee, Nigeria and South-East Asia, marketing strategies were intense and persuasive, encouraging AMU amongst farmers.^28^^,^^39^^,^^118^ In contrast, farmers in the UK were sceptical of the information provided by herd nutritionists.^35^ Farmers in South Carolina, Cambodia and Tennessee suggested that administration instructions should be simplified, and AMU information should be authentic.^54^^,^^65^^,^^103^ Furthermore, farmers in Cambodia and the UK suggested a traffic light system would be beneficial.^18^^,^^40^^,^^104^ In Ohio, vets considered meetings between producers and vets as the most informative platform. However, they recognized that this may not be feasible due to time constraints. Therefore, the provision and distribution of information via brochures are more realistic.^63^

#### Accessibility

In Central America, Africa, India, Thailand and Tennessee, farmers indicated that there was limited access to and a high cost associated with veterinary consultation, particularly for farmers in rural regions.^21^^,^^24^^,^^34^^,^^39^^,^^46^^,^^56^^,^^69^^,^^112^ Additionally, farmers in Africa and Asia can obtain antimicrobials without prescription and antimicrobials purchased are frequently of poor quality and counterfeit. However, the low cost and easy access to these antimicrobials makes them an easy option.^21^^,^^44^^,^^47^^,^^110^ Furthermore, due to the scarcity of trained professionals and limited resources in these regions, untrained professionals are more inclined to overprescribe.^23^^,^^24^^,^^46^ In Africa ‘agrovets’ and drug vendors admitted to selling antibiotics without prescription; basing treatment choice on farmer requests or symptom description.^46^ Similarly, vets were aware that they were the last resort, as farmers tried numerous treatments that were unsuccessful before seeking advice from vets.^24^^,^^46^ In developing countries, despite vets displaying a satisfactory awareness of AMR, this awareness did not influence prescribing behaviour. Other factors including accessibility of antimicrobials, affordability, lack of information and substandard hygiene were considered before prescribing treatments.^71^ This demonstrates that the rationale of prescribing reflects social, economic, investment and commercial factors.

In developing countries, there is limited access to correct, credible information and satisfactory training about prudent AMU and AMR.^21^^,^^24^^,^^50^^,^^56^^,^^65^^,^^101^^,^^110^^,^^115^ However, a lack of access to credible, reliable information is not isolated to developing countries, as farmers in the UK also reported a scarcity of communication and information available on antimicrobials.^37^^,^^81^ In Ethiopia, many of the respondents were illiterate and therefore could not read proper dispensing instructions in relation to dose and storage.^25^ Moreover, vets in India and Africa indicated training resources were not available in comparison to other countries.^46^^,^^65^ In the UK, poor availability of highly skilled stock-people was a barrier to reducing total AMU in pigs.^43^

Globally, farmers and vets report financial constraints and vulnerability on the farm, meaning that farmers are unable to invest in suitable structural improvements to facilitate recommended antimicrobial stewardship interventions.^15^^,^^17^^,^^22–24^ Furthermore, in developing countries, there is limited access to professional advice and credible sources of information. Coupled with easy access to counterfeit and substandard antibiotics and poor enforcement of policies, this creates an environment where imprudent AMU is normal behaviour and is not discouraged.^21^^,^^44^^,^^46^ While strategies to reduce AMU should come from being involved in ‘best practices’, the reality for vets and farmers is that changes must be economically viable, as demonstrated by the fact that involvement in antimicrobial stewardship practices, such as vaccination programmes, fluctuated depending on financial support.^69^ Therefore, future strategies should be designed to alleviate the financial burden felt by farmers and centre around positive incentives to support AMU reduction as opposed to penalties.^68^^,^^81^^,^^89^^,^^121^ These strategies could include reducing veterinarian tariffs, subsidized vaccination programmes and grants for investment in farming structures. In addition, professional advice and information sources must be accessible, authentic and user-friendly.^17^^,^^37^^,^^40^^,^^44^^,^^68^^,^^76^^,^^121^

### Theme 5: Factors influencing AMU

#### Habitual behavioural patterns

In the UK, Switzerland and Michigan, AMU was influenced by habitual behaviour, with farmers tending to mirror AMU behaviours from the previous year.^16^^,^^30^^,^^37^^,^^44^^,^^89^^,^^100^ In the Netherlands and Germany, extending antimicrobial treatment was socially accepted.^82^ Similarly, in Cambodia, Nigeria, Ethiopia and Ghana, habitual behaviour surrounding misuse and overuse of antimicrobials was found to be socially acceptable.^25^^,^^49^^,^^104^

#### Vet prescribing factors

##### Client pressure

The majority of vets in European countries felt pressure from clients to prescribe antimicrobials.^17^^,^^35^^,^^68^^,^^74^^,^^76^^,^^86^^,^^101^^,^^107^^,^^122^ Interestingly, Finnish and Australian vets indicated that social pressure was unlikely to influence their prescribing decisions.^89^^,^^95^ Speksnijder *et al.* (2015)^76^ reported that client pressure was prevalent in vets specializing in intensive farms. Client pressure was not isolated to European regions; vets in India and Peru also felt client pressure to prescribe antimicrobials.^65^^,^^108^ Client expectations and preferences were a common reason to prescribe antimicrobials, thus vets prescribed to keep their clients satisfied, maintain their relationships and reduce the risk of clients going to a competitor’s practice.^72^^,^^74^^,^^75^^,^^86^^,^^88^^,^^101^^,^^122^

##### Visits/farmer relationship

Studies indicated that treatment choice varies depending on individual farm characteristics such as infrastructure, overall herd health and concerns over hygiene and sanitation.^17^^,^^71^ UK studies cited the importance of regular farm visits and availability of farm records, providing insight into livestock status when prescribing antimicrobials and suggesting strategies.^17^^,^^35^^,^^63^^,^^106^ Furthermore, Doidge *et al.* (2019)^106^ indicated that vets were more likely to prescribe antimicrobials to long-standing clients and there was confidence in the farmer’s judgement of disease. Swiss and UK vets acknowledged that a farmer’s individual personality influenced prescribing behaviour. That is, vets considered the farmer’s compliance to treatment plans when deciding treatment.^17^^,^^75^ Vets in Peru emphasized that physical examination of the affected animal(s) was preferred, although vets in these regions often depended on vague descriptions of the symptoms.^108^

##### Uncertainty

Dutch, Italian, Irish and UK vets occasionally prescribed antimicrobials due to uncertainty surrounding the diagnosis, fear of blame for unsuccessful treatment, concern for their reputation and prolonged animal suffering.^17^^,^^76^^,^^106^^,^^121^ Furthermore, vets felt a burden to successfully identify the disease and achieve a rapid resolution, feeling that delaying treatment that later proved necessary encouraged prescription.^35^^,^^62^^,^^74^^,^^89^^,^^101^ Additionally, Australian vets felt there was a lack of clear guidance to treat some conditions.^89^

##### Peers/previous experience

Swiss vets’ prescribing behaviour was based on knowledge obtained from education, training and reading scientific articles.^75^^,^^117^ As previously discussed, vets rely on advice from peers and their previous experience as a source of information. Vets acknowledged reliance on the previous positive experience of treatment or the advice of peers when prescribing antimicrobials.^59^^,^^64^^,^^75^^,^^86^^,^^88^^,^^89^^,^^107^^,^^116^^,^^117^^,^^122^ Similarly, Irish vets indicated that previous experience with a specific antimicrobial, coupled with farm experience, was influential when deciding treatment.^122^ Furthermore, UK vets admitted that they are more likely to prescribe antimicrobials if another vet had done so previously.^106^

##### Time pressure and workload

Studies in Ireland, India and Australia highlighted that busy schedules and heavy workloads, can lead vets to prescribe antimicrobials without veterinary consultation.^65^^,^^89^^,^^122^ Similarly, in Australia and Ireland, vets indicated that it is easier to prescribe antimicrobials than spend time convincing a farmer that they are unnecessary or returning to a farm later when the animal’s condition deteriorates.^88^^,^^101^ Interestingly, vets in the UK were more inclined to prescribe antimicrobials to farmers if they had more time to discuss the matter with them.^106^

##### Practicalities

Several European studies indicated that ease of administration and the farmer’s ability to administer antimicrobials appropriately governed vets’ treatment choices.^65^^,^^115^^,^^117^^,^^122^ Similarly, in India and Italy, the administration route and withdrawal periods were considered, particularly for intensive sectors i.e. porcine.^63^^,^^65^^,^^107^ In other studies, non-clinical factors were considered such as cost and animal temperament.^86^^,^^122^ Vets in New Zealand and France acknowledged that individual practice habits and policies were important when it came to prescribing antimicrobials;^72^^,^^86^ while in developing countries access to and availability of antimicrobials influenced treatment choice.^71^ In the USA and Denmark, vets indicated that government regulations influenced treatment choice.^117^^,^^121^ In the UK, despite AMR awareness, vets indicated that AMR is not always prioritized in treatment choice, and they adjust their treatments to specific circumstances i.e. to preserve animal welfare and productivity, sometimes at the expense of antimicrobial stewardship principles.^17^

Overall, although treatment choices are primarily based on clinical aspects, vets have acknowledged that various other non-clinical aspects influence treatment choice.^17^^,^^76^^,^^88^^,^^106^ Therefore, it would be beneficial to implement strategies that harmonize decision-making around treatment plans.^73^

#### Legislation

Lenient AMU legislation in developing countries in Africa and Asia, coupled with easy access to antimicrobials from illegal, poor quality or counterfeit drug vendors provides an environment for imprudent AMU.^22^^,^^24–26^^,^^28^^,^^104^^,^^112^ Furthermore, in China, farmers were able to purchase antibiotics without a prescription or consultation.^84^ Poor enforcement of policies was evident as farmers in Asia and Africa were unaware of the consequences of violating antimicrobial regulations and were rarely reprimanded for violating regulations.^20^^,^^46^^,^^49^^,^^123^ Additionally, farmers indicated that even if they were aware of regulations, the majority would not adhere to current recommended practices.^20–22^^,^^103^ Furthermore, farmers in South-East Asia were reluctant to implement new legislation, policies and strategies to reduce antimicrobials.^28^^,^^103^ In India, however, legislation strengthening surveillance to promote rational prescribing and reduce substandard AMU has been successfully implemented to mitigate AMR.^65^ Overall, studies have identified that introducing policies and legislation to restrict AMU could result in recoil from farmers (as they do not feel supported) and lead to unintended consequences, such as unregulated AMU, as farmers feel that it is necessary.^43^^,^^60^

#### Laboratory testing

Although American vets were uncertain that laboratory testing was necessary to diagnose disease, ^30^ the majority of vets believed laboratory testing (diagnostic and susceptibility) is an effective initial approach to correctly diagnose and treat disease, reassuring vets on treatment choice and educating farmers, thereby reducing the emergence of AMR.^64^^,^^65^^,^^68^^,^^72^^,^^116^ Despite the obvious benefits of laboratory testing, it is not common practice on farms, as farmers and vets feel that it is impractical due to the increased cost and time associated with testing and delayed treatment action. For these reasons, vets found it difficult to convince farmers to perform tests, as farmers wanted animals treated quickly.^17^^,^^24^^,^^65^^,^^68^^,^^88^^,^^101^^,^^116^ In Australia, laboratory testing was only used for cases with persistent recurring infection, severe disease and/or a specific disease location, as most vets thought that diagnostic and susceptibility testing led to the overuse of antimicrobials.^88^ De Briyne *et al*. (2013)^116^ reported that vets used laboratory testing if there is a poor response to initial treatment. In Tennessee and Ethiopia, the majority of respondents never used laboratory testing for diagnosis.^54^^,^^113^ In contrast, the majority of vets in Denmark, Finland and Sweden stated that they frequently or always use laboratory testing to diagnose mastitis, in comparison with only 18% of Norwegian veterinarians.^95^^,^^117^ Furthermore, in Switzerland, 43% of farmers let their vet decide whether to complete antimicrobial testing.^42^ In India, farmers and vets believed these facilities were poorly set up and did not provide adequate information.^24^^,^^65^ Meanwhile, European vets and farmers sought validation of test effectiveness and tests to be regulated by external government bodies.^116^

Overall, undoubtedly, laboratory testing is an effective tool to alleviate AMR. However, it is not popular with farmers and vets due to perceived costs, delayed results, delayed treatment and uncertainty and ambiguity over effectiveness and credibility. Therefore, information should be provided to farmers and vets to provide clarity. In addition, work should be done to improve the mechanisms of laboratory testing to lessen result waiting times, making this tool more appealing and suitable for farmers and vets.

#### Current practices amongst farmers and vets

In Australia and New Zealand, the majority of vets did not have policy documentation within the practice.^86^^,^^88^ In Ohio, while vets were confident that the majority of farmers followed protocols, only 23% of vets provided farmers with protocols.^63^ Conversely, in Switzerland >80% of vets provided farmers with verbal or written protocol information.^42^ Furthermore, in Australia, vets admitted to using HP-CIAs weekly, while vets in Italy admitted to using HP-CIAs as the first choice of treatment.^62^^,^^88^ In India, the majority of vets and farmers never discussed previous experiences of disease treatment.^65^ In the UK and Italy, prophylactic AMU remains a preferred method to treat common issues.^62^^,^^64^ In addition, vets in the UK revealed that despite the ban to use antimicrobials as growth promoters, on occasion they are still used for this purpose.^64^

Unequivocal evidence has indicated that globally imprudent AMU is evident at varying degrees of severity.^40^^,^^44^^,^^56^ In Cambodia and Turkey, antimicrobials were used prophylactically and for growth promotion even though this practice is prohibited.^32^^,^^58^^,^^104^ In the UK, over half of the participants used antimicrobials to prevent disease.^43^ In Canada, Asia and Africa, farmers only sought veterinary advice after several unsuccessful treatments are attempted.^23–33^^,^^58^^,^^104^^,^^123^ Meanwhile, studies in Central America, China, Cambodia and Thailand reported that farmers frequently use multiple antibiotic treatments simultaneously in a prophylactic manner,^20^^,^^22^^,^^49^^,^^50^^,^^104^^,^^112^ with farmers in Canada and South-East Asia reporting that frequent, unsupervised use of HP-CIAs was common.^28^^,^^33^^,^^107^ While, studies in Malaysia, India, China, Sweden and the UK revealed storing antimicrobials was common practice.^15^^,^^16^^,^^52^^,^^53^^,^^84^ Furthermore, antibiotics were frequently misused without a proper prescription, dose, frequency, duration (whether too short or too long) and route of administration in Ethiopia, India and Bangladesh.^24–26^,^32^^,^^61^^,^^110^^,^^113^^,^^114^^,^ Moreover, in Thailand and Sweden extending antibiotic treatment was perceived as a thorough approach to maintain herd health,^22^^,^^82^ while farmers in New York administered a greater dose of antibiotic than recommended by the directions on the label if they felt they were treating a more serious illness because they believed it would help the animals’ recovery.^34^ In Canada, Ethiopia and Sudan, farmers noticed that antimicrobials had become less effective.^25^^,^^26^^,^^33^ Farmers in the UK provided the milk with antibiotic residues to calves.^40^ Varying compliance with withdrawal periods, record keeping and disposal was apparent across numerous countries globally.^20–24^^,^^31^^,^^41^^,^^54^^,^^61^^,^^65^^,^^70^^,^^92^^,^^123^^,^^124^ In Bangladesh, Nigeria and Ethiopia, farmers engaged in what is considered poor farm practices and disinfection.^44^^,^^113^^,^^114^ In Michigan, the majority of farmers practised good farm management practices and had a vaccination programme.^30^

To summarize, this review has identified that the factors influencing farmers’ and vets’ behaviour in relation to AMU are multifaceted and complex. As discussed, farmers’ and vets’ decision-making are based on a delicate balance of knowledge, finances, productivity, animal welfare, attitudes, perception of disease risk and strategies, concern for AMR, access to resources and habits. Moreover, it is evident both farmers’ and vets’ motivations and justifications to make decisions on AMU and antimicrobial stewardship strategies are based on a continuous personal evaluation of these factors. Subsequently, addressing only one of the aspects discussed when designing interventions is not enough to encourage behaviour change. In actuality, incorporating the different factors discussed into an intervention provides a holistic approach, which would be more promising in encouraging responsible AMU and mitigating AMR.

## Recommendations for future research and strategy design

Owing to the huge variability in relation to knowledge, attitudes, perceptions and behaviours of AMU and AMR reported in this review, an initial assessment for each sector or country is essential to establish current attitudes. An approach of this nature will identify specific priority areas and gaps in knowledge and skills for farmers and vets; this will then enable tailor-made strategies to be developed. Strategies should encourage incremental behaviour change so that farmers and vets feel capable of implementing antimicrobial stewardship practices. Additionally, strategies should preserve production, safeguard profit and maintain/improve animal welfare simultaneously, thus supporting and empowering farmers and vets to change behaviour.

Nordic regions are considered the ‘gold standard’ or global leaders modelling prudent AMU. In Denmark (2010), the Yellow Card initiative was launched by specifying a threshold of AMU in relation to animal daily dose per 100 animals a day; when antimicrobial consumption exceeds the threshold, annual veterinary inspections are increased for 5 months.^77^ In addition to improving overall health, Norwegian strategies included assigning one contracted vet per herd with mandatory veterinary inspections and clear reduction targets for livestock production.^76^ Similarly, in Switzerland a Strategy on Antimicrobial Resistance (StAR) was launched, focusing on educational and training programmes,^77^ whereas in the Netherlands strategies included improving herd health with mandatory herd health plans, assigning clear responsibilities in herd health management and prescription/delivery of antibiotics. The strategies mentioned above vary in approach; however, all of them have been successful in significantly reducing AMU.^76^^,^^77^ Meanwhile, the Dutch government in 2010, stipulated that the livestock sectors must reduce their AMU by 50% in 2013 and 70% in 2015, in comparison to AMU figures recorded in 2009. After an initial rapid AMU decrease on farms (56%) in 2013, antimicrobial reduction plateaued (at 58%) in 2014. To satisfy the targeted 70% reduction, additional changes in the behaviour of vets and farmers towards AMU in livestock sectors were required.^76^ This emphasizes that behaviour change in relation to AMU needs continuous evaluation to generate innovative concepts that are desirable and feasible to farmers and vets to promote responsible AMU. Therefore, identifying and understanding the psychological factors (barriers and facilitators) in relation to AMU is essential, and incorporating these factors into intervention design will increase desirability and acceptability from key stakeholders, improving intervention success.

The overall narrative of the articles included in this review is to reduce AMU. While reduction of AMU is crucial, vilifying antimicrobial usage is dangerous, as antimicrobials are essential to preserve animal welfare. The ramifications of this type of narrative promotes the adoption of unrealistic and uninformed marketing strategies; for example, recent research has reported an increased frequency of ‘raised without antibiotics’ marketing campaigns within food-producing sectors. While it is essential to reduce AMU, a better approach may be to focus future research on responsible and safe AMU.

## Strengths and limitations

This research has a number of strengths and limitations. To the best of our knowledge, this critical review is the first to explore farmers’ and vets’ knowledge, attitudes and perceptions in relation to antimicrobials and AMR, across all food-producing sectors on a global scale. The review collated, synthesized and compared findings from a range of study designs (including surveys, interviews, focus groups and a mixed-method approach), from multiple countries worldwide, forming an overall picture of the factors that influence behaviours in relation to AMU, ultimately identifying common barriers and facilitators. Thus, the findings from this review can inform strategy design aimed at encouraging behaviour change to promote responsible AMU, mitigating AMR. Despite the inclusion of a large number of studies (*n* = 103), the vast majority were completed in Europe, which limited the generalizability of the results. Furthermore, the distribution of studies relating to different food-producing sectors was not even, with most research being carried out in the dairy sector and a paucity of research in the sheep sector. Finally, studies included in this review spanned the last two decades and thus may not fully reflect current knowledge, attitudes and behaviours towards AMU.

## Conclusions

There is huge variation in knowledge, attitudes and practices across countries, production types, sectors and individual farms, highlighting the complexity of reducing AMU globally. Studies have shown that increasing knowledge and awareness of AMU can positively influence attitudes and interest towards implementing strategies. However, while increased knowledge and awareness is beneficial it is not always enough to reduce AMU. Farmers have acknowledged that several other barriers affect their ability to implement strategies, such as financial constraints, labour requirements, access to resources, perceptions about capabilities and feelings of uncertainty. In European countries, fostering a collaborative relationship between vets and farmers is necessary to elicit a shared ownership to combat AMR. In developing countries, drug vendors and peers are viewed as trusted and accessible sources of information. Therefore, strategies targeting these groups would be an effective approach for interventions surrounding awareness. Behaviour change theories should also be included in strategy design, to address barriers experienced in agriculture in relation to AMU. Overall, careful consideration and an evidence-based approach is required to develop optimized intricate interventions and strategies, which elicit successful and sustained behaviour change.

## Funding

This work was supported by *safe*food, the Food Safety Promotion Board, under Grant No. 04-2018.

## Transparency declarations

None to declare.

### Author contributions

C.M.K., M.D. and T.B. were involved in the conception of the review; C.M.K. conducted the majority of literature searches and drafted the manuscript and authors T.B., S.F. and M.D. edited it. All authors read and approved the final manuscript.

## Supplementary data

Table S1 is available as Supplementary data at *JAC-AMR* Online.

## Supplementary Material

dlab178_Supplementary_Data
