# Supplementary material for: Antimicrobial use in agriculture: critical review of the factors influencing behaviour
Source: JAC Antimicrob Resist. 2021 Nov 30;3(4):dlab178. doi: 10.1093/jacamr/dlab178 (PMC8634307; doi:10.1093/jacamr/dlab178)
Supplement: dlab178_Supplementary_Data [file dlab178_supplementary_data.docx]

**Supplementary data**

**Table S1**. Comprehensive summary of key themes and sub-themes identified in this review

| **Themes** | **Sub Themes** | **Quantitative Studies (n=)** | **Qualitative Studies (n=)** | **Studies Mixed method (n=)** | **Total Studies (n=)** | **Interpretive Quote** | **References**  **(**Quantitative**, Qualitative,** *Mixed methods)* |
| --- | --- | --- | --- | --- | --- | --- | --- |
| **Knowledge and Awareness** | Developing countries display limited knowledge of antimicrobials usage and risks | 18 | 2 | 3 | 23 | " There is a lack of knowledge and awareness as only 45% of farmers were able to correctly identify AMR."  "Vast majority of farmers has little or no knowledge of the risk of using antimicrobials"  "Most farmers indicated lack of knowledge on why treatment failure had happened and did no relate his to possible AMR."  "Overall farmers level of awareness was partially knowledgeable." | 20-23, 25, 27, 49-50, 52-53, 57-58, 61, 84, 110, 113, 114, 115  **56,** **124**  *26, 28, 44* |
|  | Developed countries display good knowledge of the purpose of antimicrobials and risks, however ambiguity persists. | 8 | 6 | 3 | 17 | "Overall farmers appeared knowledgeable about the prevention and treatment of common diseased in dairy herds e.g., mastitis."  "Farmers were shown to be coherent in their choices of treatment"  "86.1% of farmers reported that they were aware of AMR, however when asked to describe AMR , only 55.4 % provided an accurate description"  "Most farmers had a decent understanding of AMR, however there was also evident there was some confusion and uninformed farmers." | 15, 29; 31; 32-33; 40-42  **14, 16, 34, 36-38**  *17, 30, 43* |
|  | Knowledges in relation to production type, herd size, gender, education level and experience | 13 | 0 | 3 | 16 | "Mean knowledge scores were higher for dairy farmers in comparison to veal and pig farmers."  "Larger farms were either more aware of biosecurity risks of more able to undertake preventative measures because the frequency of diagnostic testing and examination of purchased cattle increased with her size in comparison to smaller herds." | 23, 32-33, 40, 45, 47-54  *44,46,65* |
|  | Vets’ knowledge and Awareness | 6 | 9 | 5 | 20 | "Study found that most of the clinicians were aware of the fundamental clinical aspects of antibiotic resistance, i.e., the general causes and transmission of resistance, response during treatment failure."  "Some respondents indicated that vets need to improve knowledge on AMU and AMR" | 59, 66, 68, 70, 73, 117  **16, 64, 67, 69, 71, 72, 74, 76, 101**  *62, 63, 65, 88, 96* |
|  | Improving knowledge as a strategy to promote prudent AMU | 20 | 12 | 7 | 39 | "Farmer workshops may be beneficial as farmers that have had positive experience with SDCT can share knowledge which may encourage other farmers to go down the same route."  "Courses in continuing veterinary education (CVE) and farmers is essential"…  "vets thought that exposure to education followed by self-directed education via webinars or podcasts, additional education indicated willingness to change behaviour”  "knowledge was the most influential factor significantly inversely related to AMU across all livestock sectors. "Majority of farmers displayed interest in deepening their knowledge of antimicrobial resistance" | 15, 20, 21, 23, 29, 31, 32, 36, 40, 41, 45, 48, 54, 57; 58, 59, 61, 66, 68, 73  **16, 37, 39, 55-56, 72, 74-76, 81-82,20 101**  *26, 28, 43, 44; 60, 63, 65,* |
| **Attitudes Towards Antimicrobials** | Farmer and vet responsibility | 6 | 12 | 4 | 22 | " Farmers felt great satisfaction for personally caring for the animal... felt responsibility to their animals" "Farmers believed that extending treatment was a 'thorough' approach and associated with good stockmanship and strengthens the farmers pride within their peers."  "Farmers had a clear perception of what a good farmer is- someone who takes care of their animals is and they strive to be recognised as such by other farmers. "  "High producing cows often described by farmers as creating a special emotional bond with farmer."  "Ensuring optimum pig health and welfare was maintained was describes as a driver for AMU by vets and considered a moral obligation"  "When farmers did not follow veterinary advice, vets felt obliged to treat sick animal and alleviate it suffering."  "Farmers believe they were not responsible for monitoring antimicrobial usage" | 20, 42, 68, 83-84, 107  **14, 16, 35, 38-39, 55, 64, 74, 76, 81, 82, 85**  *28,43, 65,* *86* |
|  | Farmer and vet Optimistic Bias | 14 | 10 | 3 | 27 | " In general, farmers perceived their own AMU to be lower than their peers in the same country and lower or similar to that of farmers in other countries."  "AMR on farms, farmers were defensive and believed that they prudently used AM, and other farmers do not, farmers frequently increasing AMR is more likely use to human imprudent AMU"  "Most participants believed that they used AM in a responsible manner, however a minority of mixed-species vets did not share this opinion and believed that pig focused vets could over prescribe AM with the use of long courses of in-feed AM for disease prevention and may not be an effective management tool" | 21, 29, 30, 31, 33, 51, 54, 59, 68, 83, 89-90, 98, 33 **16, 34-35, 39, 56, 64, 69, 82, 85, 82**  *65, 86, 88* |
|  | Individual perception and attitude towards disease risk and strategies | 32 | 12 | 9 | 53 | "Farmers emotions and personality are associated with differences in farmer management Further understanding how personality and emotions influence change in behaviour is key to increasing uptake of new information."  "Different attitudes towards treatment may reflect how strongly a farmer is attached to their animal, as cow-oriented farmers are more likely to extend treatment more frequently in comparison to business-oriented farmers."  "Attitudes to disease risk measures seem strongly linked to attitudes to disease risk itself. Many respondents base their decisions on implementing specific practices on their personal assessment of trade-off between risk, efficiency and cost."  "Differences between sectors are marginal, also there seemed to be more or a difference at an individual farm level in comparison to livestock sectors. But dairy farmers did display more of a disregard in comparison to others were more inclined to use AM without consulting advice from the vet."  "Interviews show that farmers compliance with recommended practices is strongly influenced by attitudes to disease risk."  "Several referred to luck- 'keeping fingers crossed' and 'touching wood' and the sense of doing what one normally does, only more intensively."  "The influence veterinarians potentially have on the attitude of farmers and the variability found in their attitude and behaviour, veterinarians need specific attention if regional or national programs are organized trying to change behaviour of farmers and encourage prudent AMU and SDCT."  "Multivariate analysis of covariance indicated that farmers from the 4 countries differed significantly in their perceived risk of and need from AM as well as in perceptions of information provided to by farmers. Belgian farmers perceived more risk from AM in comparison to German farmers. Belgian and Swedish Farmers perceived more need to use AM in pig farming than in France. Swedish farmers though that their vets provided more information in comparison to German and Belgian farmers."  "Perceived risk: Reflected the perceived risk towards AMR at the farmers personal level, their farmers, ad public health. Overall pigs and veal perceived a higher risk of AMR in comparison to dairy farmers" | 15, 21, 29, 32, 33, 40, 41-42, 47-48, 50, 51, 57-59, 61, 66, 68, 73, 83, 89, -95, 97-100, 114  **14, 16, 34, 36, 37-38, 55, 74, 76, 81- 82, 101**  *30, 43, 44, 60, 65, 86, 88, 93, 96* |
|  | Farmer and vets’ attitudes towards reducing AMU | 37 | 15 | 6 | 58 | "42% of farmers were strongly intended to reduce AM. 70% of respondents agreed with the statement that reduced use of AB would be a good thing to do. Only 60% of farmers believed they have the skill set and knowledge to reduce AMU"  "Farmers suggesting that they would not be able to reduce antimicrobial use on farms"  "The perceived benefits and the information provided by vets was relatively high in four European countries.  "The majority of vets agreed that it is important that AMU in livestock production is restricted and 89% of vets believed they can still be a good vet when they use less AM."  "Adverse reactions have occurred with intervention…. Many vets expressed concerns with the switch from BDCT to SDCT, due to misclassifying infected cows and hence failing to treat them which may result in the fatality of a ruminant. Therefore a sense of responsibility to the health of the animal and economic aspect for farmer and reputation for vet as well"  "Farmers were worried that decreasing AM and uncertain on the impact of animal health and welfare would be compromised."  "Farmers perceived a lot of benefits from the application of AM, although they do not believe AM were absolutely necessary to keep pigs healthy…Farmers may consider AM as highly effective and useful but also recognise that there are other methods to produce healthy pigs without relying on antimicrobials."  "91% of farmers believed that their profitability would decrease if they could not use injectable antimicrobials. > 33% of producers reported that their profitability would decrease their profitability in feed and water."  "Majority of respondents felt that AMU is the most important factor for achieving disease protection and 64% believed that the primary reason AM were used on the farm were for economic cost benefits and 37% believed that the continued non-therapeutic use of AM did NOT result to AMR"  "Pig farmers believed that Antimicrobials cured and prevent ed farm diseases and improved overall herd health” | 15, 20, 22-23, 29, 31-33, 40-42, 45, 48-52, 54, 57-59, 61, 68, 73, 83, 90- 92, 94, 97-100, 102, 105, 110, 114  **16, 24, 34, 35, 37, 38-39, 55-56, 64, 67, 74, 82, 85, 104**  *30, 43, 44; 63, 88, 103* |
| **Influential Relationship** | Vet/farmer relationship | 21 | 17 | 6 | 44 | "Vets are seen as the most credible and reliable source on disease and disease risk management, providing more farmer-focused advice than government"  "The perceived benefits and the information provided by vets was relatively high in all four European countries."  "Veterinarians were perceived as the most trusted information source."  "88% of vets proactively encouraged farmers to reduce AM and believed the advice they provided farmers regarding SDCT was the best possible approach. >Therefore, most farmers were positive about the implementation of the policy brought about in 2012 to limit the use of AM in preventative form. Vets attitudes are important as they can influence the farmers perceptions as well"  "Communication and understanding between farmers and vets is essential."  "Vets mentioned that in addition to their curative work, providing advice to farmers is increasingly becoming a part of daily work. The advice focuses on promoting animal health and welfare and improving production results."  "There is movement to vet is moving towards and advisory role focused on improving and maintaining heard health rather than treat sick animals."  "Vets were comfortable on the topic of AMR, however only 23% of vets routinely discussed AMR with clients.... 77% felt their clients followed protocol, with only 23% of vets admitting they supply the correct protocol to the client" | 15, 21; 29; 32-33, 40-42 50, 59, 68, 73, 83, 90- 91, 95, 97-98, 106-107, 110  **14, 16, 35-38, 55, 64, 72, 74, 76, 82, 108,** **81-82, 101, 108**  *30, 44, 63, 65, 86, 96* |
|  | Peers and Previous experience | 17 | 14 | 7 | 38 | "Peer experience and experience from other sources e.g., feed nutritionists were a trusted source of information."  "> "Peer support and previous experience are important in regions where vets are difficult to contact."  "Farmers also practice indigenous knowledge and use alternative (homeopathic remedies) to treat diseases"  "In Sudan a poorly educated group of farmers knowledges is acquired while working on farms and personal experiences and exchanges and shared with each other."  "Majority of farmers were raised on farmers, therefore relied on years of experience when deciding frequency and dosage of AM in sick cows"  "Vets normally depend on advice of peers or previous experience when prescribing AM."  " Interestingly, a minority of participants acknowledges that senior colleagues may pressure on junior participants on AMP to maintain good relationships with long-standing clients and practices."  " With age vets perceived to have increased confidence with prescribing behaviour."  "Years of veterinary experience negatively correlated with and factors withing knowledge surrounding AMR and may be due to the focus of AMR and AMU in the curriculum of recent graduates.” | 15, 23, 32, 50, 54, 58-59, 68, 83, 86, 106, 110, 113-117  **16-17, 24, 39, 55-56, 64, 74, 81-82, 101, 104, 111-112**  *26, 30, 63, 86, 88, 109, 118* |
|  | Government / Society | 4 | 10 | 3 | 17 | "Society (government, regulators and policy makers) was perceived as the only negative reference group"  "Politics and regulation all farmers were unhappy and defensive about how their production is portrayed in the media and felt a lack of appreciation from society of their work."  "Advise from vet is more credible than government bodies, as farmers suggested government bodies have no idea indicating that it was unnecessary or impractical to adopt one or more of the proposed measures recommended by government bodies... and they do not have faith in government bodied to help" | 20, 21, 89, 105  **14, 16, 34-35, 37, 39, 64, 67, 76, 82,**  *28, 43, 88* |
| **Resources** | Finance | 20 | 16 | 9 | 45 | "In addition AM were considered a cheaper short term to a disease intervention in contrast to the much higher cost of upgrading farming environment and improving environment even though these factors were perceived in reducing overall AM requires on farms"  "All respondents mentioned that economic considerations were a major driver for decisions made in farming, cost control via low-quality feeds, postponing costly improvements infrastructure and farm staff were mentioned, low-quality livestock arriving at farms which result in increased sensitivity to disease."  "Other vets mentioned the difficulties related to predicting the cost effectiveness of specific measures in the context of a specific farm, and it was difficult to convince farmer of necessity to implement specific measures"  "Vets in this study believed that the profit margin for any AM would motivate prescribing behaviours, and decoupling od AM sales where vets are no longer able to dispense AM would not influence the amount of AM used...EU has indicated that they may decouple the AM sales from vets to eliminate potential profit drive prescribing behaviours."  "All respondents stated that pharmacy incomes from AM selling were no longer a stimulus, some vets indicating incomes are replaced by vaccines and non-steroids."  "were defensive and strongly disagreed that profit from AM sales influences their decision to prescribe" "economic benefits were low when it came to prescribing and AM other factors are considered first"  "Tight profit margin and economic vulnerability of these livestock sectors have been shown to be drivers of AMU to ensure farm profitability, disease prevention and reduce mortality. " | 12, 15, 21-22, 27, 29, 41, 49, 51, 58, 66, 68, 83, 90-91, 97-99, 115, 121  **14, 16, 17, 24, 34-37, 64, 69, 72, 76, 81-82, 101, 104**  *28, 30, 43, 44, 46, 65, 86, 88, 96* |
|  | Information sources | 11 | 12 | 10 | 33 | "Despite electronic information being easily available, vets preferred information via one-to one meetings between vets and producers as an effective means to educate on AMR. However, this was also suggested to be unrealistic as due to time-constraints of the job. Therefore vets agreed that the distribution of handouts containing management practices and diagnosis description and dosage guidelines would be more realistic and feasible"  "Often misleading, incorrect or imprecise labelling information on feed as in south east Asia is routinely added to feed to prevent disease in livestock."  "Many of the respondents were illiterate, therefore could not read proper dispensing instructions in relation to dose and storage."  "Farmers indicated that there was a lack of information available to farmers about prudent antimicrobial use and contamination in the environment" | 15, 21, 33, 40, 54, 58-59, 83, 90-91, 117  **17, 24, 35-37, 39, 64, 75, 81-82, 101, 104**  *28, 30, 43-44, 60, 62, 63; 65, 88; 109* |
|  | Accessibility to resources | 24 | 14 | 9 | 47 | "According to vets there is probably an access to cheaper adulterated or counterfeit drugs"  "Farmers has access to illegal drug vendors, dispensing counterfeit and AM under unfavourable conditions e.g. direct exposure to sunlight."  "Farmers were able to access antimicrobials without prescription"  "Results also indicated that vets were less exposed to training resources in comparison to other countries."  "The main challenge affecting the correct application of AM was lack of adequate sources of information especially in relation to relevant training followed by a lack of publicity about the available sources" | 15, 20-23, 25, 27, 32-33, 40, 49, 50, 52, 54, 58, 61, 84, 92, 99, 110, 113, 114-115, 123  **14, 24, 34-37, 39, 56, 69, 71, 81, 124, 108, 112, 124**  *26, 28, 30, 43-44; 46, 60, 65, 103* |
| **Factors influencing AMU** | Habits | 6 | 4 | 2 | 12 | "Almost 59% of respondents reported that their AMU was similar to previous year" | 15, 25, 49, 90, 100, 113  **16, 37, 82, 104**  *30, 103* |
|  | Vets Prescribing factors | 10 | 11 | 7 | 28 | **Client pressure**: "The majority of respondents felt the pressure from clients to prescribe. However, 25% of intensive specialists felt pressure to prescribe every 2 months."  "Firstly, vets feel pressure to keep vets happy because of competition among prices and fear clients would consult a new practice if they were displeased with the service."  "Vets sometimes consider the demand of farmers for AM"  **Vet/Farmer relationship:**  "The farmer wants/expects AM... Vets are likely to prescribe AM to develop p or maintain client relationships."  "Farmer-vet relationship was also influential as vets are less likely to prescribe to new clients in comparison to a long-term client. Suggesting that long-term relationship increased the vets likelihood to prescribe"  **Uncertainty:** " Vets are not confident in your diagnosis'  **Peers/Previous experience:** "Individual practitioners tended to work independently of these and sought information from own experiences, history of the farm and peers if required colleagues was a trusted source"  **Time pressure/ Workload:** "Long working day and lack of time, it was easier to dispense AM without spending time convincing clients the AM were not necessary."  "The results of the study identified that vets are more likely to prescribe AM to farmers if they have more time to discuss the matter with them"  **Other practicalities:** "non-pharmaceutical factors considered were, cost, animal temperament, your experience using that AM on the farm and the farmers ability to administer the AM."  "Ease of administration was considered by 25% of vets and vets agreed that 75% of vets believed that the withdrawal period was key" | 59, 68, 89, 95, 106-107, 116-117, 121-122  **16, 35, 62, 64, 71-72, 74-76, 101, 108**  *30, 43, 44; 63, 65, 86, 88* |
|  | Legislation | 12 | 10 | 5 | 27 | "Majority of farmers had no knowledge punishment for violating laws, and were rarely penalised for violating laws"  "Stricter legislation surrounding AMU may seem an obvious aid combating misuse of AM, however research has suggested that this may only increase the application and sourcing of black market AM."  "policy measures that would prohibit the drug sales by vets was seen as least effective."  "Some farmers believe external pressures such as legislation should be a last resort as a vet said 'the ultimate one would be legislation, but I would prefer if it didn’t get to that stage. I prefer to use a carrot rather than a stick." | 20-22, 25, 27, 49, 52, 83-84, 99, 115, 123  **14, 17, 24, 34-37, 69, 104, 112**  *26, 28, 44, 46, 60* |
|  | Laboratory Testing | 6 | 6 | 3 | 15 | "Vets have a legal basis for explaining to farmers that they can’t give CIA's without additional tests"  "Lack of research in AM testing was also identified...Requirement for more rapid results from the lab and several factors influence choice of lab including, proximity, availability, responsiveness and expertise...laboratory reports could be improved so vets can train and educate farmers in AMU" | 42, 54, 95, 114, 116-117  **16, 24, 64, 72, 76, 101**  *30; 65, 88* |
|  | Current AMU practices | 23 | 8 | 12 | 43 | "Farmers frequently stored antimicrobials and were unaware this was a poor practice and actually thought it was good practice "  "76% of farmers indicating that they use a combination of veterinary and human antibiotics."  "Vast Majority of farmers reported noncompliance with withdrawal compliance, overdosing and using human antibiotics" | 15, 20-23, 25, 31-33, 40-42, 49; 50, 52-53, 54, 58, 61, 84, 92, 110, 113-115, 123  **14, 34, 56, 64, 82, 104, 112, 124**  *26, 28, 30, 43-44, 62-63; 65, 86, 88; 103, 109* |
